# Relevance of polymorphisms in TLR2/4 genes and their association with plasma cytokines for schizophrenia

**DOI:** 10.1038/s41435-026-00383-5

**Published:** 2026-02-26

**Authors:** Saahithh Redddi Patlola, Aodán Laighneach, Derek W. Morris, Brian Hallahan, Colm McDonald, John P. Kelly, Gary Donohoe, Declan P. McKernan

**Affiliations:** 1 Pharmacology & Therapeutics, School of Pharmacy & Medical Sciences, University of Galway, Galway, Ireland; 2 Centre for Neuroimaging, Cognition and Genomics (NICOG), University of Galway, Galway, Ireland; 3 School of Biological and Chemical Sciences, University of Galway, Galway, Ireland; 4 School of Medicine, University of Galway, Galway, Ireland; 5 School of Psychology, University of Galway, Galway, Ireland

**Keywords:** Disease genetics, Neuroimmunology

## Abstract

Schizophrenia is associated with altered levels of plasma cytokines. Toll-like receptors (TLRs) can be responsible for cytokine synthesis. It remains unclear whether genetic changes in TLRs, e.g. single-nucleotide polymorphisms (SNPs), account for altered levels. Here, we investigate the relationship between SNPs in TLR2 and TLR4 genes, cytokine levels, and cognition. This study included 281 participants (190 controls and 91 schizophrenia patients). Cognitive tasks using CANTAB and WAIS-III were administered to participants. Genotyping was performed using the Axiom-PMRA-R3 platform. Data were handled using Plink.V.1.9. We used ELISA to quantify plasma levels of cytokines (Interleukin-6 (IL-6), IL-8, IL-10, and Tumor necrosis factor (TNF-α)) and TLR activity as indicated by cytokine release from whole blood stimulated with TLR-2, -3, and -4 ligands. Linear regression of clumped SNPs revealed one TLR2 SNP [rs115751519] was associated with plasma IL-8 levels in patients. Four TLR4 SNPs [rs10983755, rs1927906, rs5030711 and rs57863323] were associated with plasma levels of IL-6 & TNF-α in patients. Three TLR4 SNPs [rs4986790, rs1554973 and rs5030711] were associated with IL-6 levels in controls. Finally, no significant associations were observed with cognition. Patients possessing a specific TLR2 or TLR4 genotype may be predisposed to elevated levels of cytokines but have no association with cognition.

## Introduction

Schizophrenia is a chronic psychiatric condition with a complex aetiology [1]. Twin studies reveal a high degree of heritability [2, 3], suggesting that genetics play a crucial role in its development [4]. Moreover, genome-wide association studies (GWAS) report multiple genetic variants and loci associated with the risk of developing schizophrenia including genes related to the immune system [5–7]. The immune hypothesis of schizophrenia is long-standing, and multiple studies have proposed a range of potential contributing environmental factors, such as maternal immune activation [8, 9] and childhood trauma [10, 11] that can induce neuroinflammatory responses. There is evidence of immune dysregulation in schizophrenia, where increased levels of proinflammatory cytokines [12, 13], increased neutrophil-lymphocyte ratios [14, 15], and neuroinflammation as indicated by neuroimaging (PET) studies [16, 17] have been reported. Additionally, associations of cytokines with schizophrenia risk have been identified in Mendelian randomisation studies [18, 19].

Genetic studies also report that polymorphisms of Toll-like receptors (TLRs) are associated with an increased risk of schizophrenia, changes in specific immune components, and cognitive performance. Notably, single-nucleotide polymorphisms (SNPs) in *TLR2* and *TLR4* have shown multiple associations with the risk of schizophrenia [20–24]. These TLRs belong to a superfamily of pattern recognition receptors that identify molecular patterns associated with pathogens and patterns associated with internal damage to the host either due to cellular stress, internal or external injury. A downstream signalling cascade is initiated in response to these molecules, leading to cytokine synthesis [25, 26]. These cytokines could further affect the developing neurons during foetal development or adolescence stage, damaging the synapses and causing cognitive deficits [27]. Cytokines [28–30] and the complement system [31] have been associated with risk of schizophrenia previously. DNA methylation studies also indicate that epigenetic changes are associated with altered cell counts of neutrophils, lymphocytes and T cell activation pathway in schizophrenia [32–34].

In our previous study, we observed elevated plasma levels of IL-6, IL-8, IL-10 and TNF-α and higher TLR2 and TLR4 activity in patients, some of which were associated with specific cognitive deficits [35]. Therefore, in this study, we investigated, if increased levels of cytokines in patients were linked to SNPs in TLR genes. We hypothesise that SNPs in *TLR2* and *TLR4* are associated with altered cytokine expression. In addition, in a Korean population, *TLR2* SNPs were associated with poor concentration in schizophrenia [36]. Therefore, we also hypothesise that SNPs in *TLR2* and *TLR4* are associated with changes in cognitive performance.

## Materials and methods

### Participants

A total of 300 participants of Irish ancestry were originally recruited for the iRELATE (Immune Response & Social Cognition in Schizophrenia) study of which 281 were eligible for inclusion in this study for final analysis. The participants included 190 healthy individuals and 91 stable outpatients, who were recruited from the local outpatient clinics and mental health services in Dublin and Galway regions. The patients were clinically diagnosed with schizophrenia or schizoaffective disorder using the SCID (Structured Clinical Interview for Diagnostic and Statistical Manual of Mental Disorders-IV). Individuals were included if they: (1) were between the ages of 18 and 65; (2) did not currently misuse substances for at least a month; (3) had a diagnosis of schizophrenia or schizoaffective disorder as defined by DSM-IV and excluded if they had: (1) a chronic inflammatory condition; (2) used non-steroidal anti-inflammatory drugs (NSAIDs) within the last 24 h; (3) a history of acquired brain injury resulting in loss of consciousness for more than one minute; (4) Any psycho-active substance use within six months prior to the study; (5) an intellectual disability (IQ ≤ 70); or (6) a diagnosed neurological disorder (e.g., epilepsy). Healthy controls (HC) were included if they additionally met the following criteria: (1) no history of psychiatric or neurodegenerative disorders; (2) no current general health issues (infections or diseases); and (3) no first-degree relatives with schizophrenia spectrum disorders. All participants provided written informed consent for the study in accordance with the guidelines set by the University of Galway research ethics committee, the clinical research ethics committee at Galway University Hospital (CA1441), Tallaght University Hospital (AMNCH), and St James’s Hospital in Dublin (2015-03).

### Blood collection

#### Cytokine quantification from plasma

Venous blood was collected from controls and patients in 10 mL EDTA tubes (Catalog # BD367873) for plasma at approximately 9:00 AM for every participant. EDTA tubes were centrifuged at 1200 g for 10 min at room temperature, and the plasma from the supernatant was collected and stored at −80 °C for further analysis. ELISA was used to quantify plasma levels of cytokines (IL-6, IL-8, IL-10 and TNF-α) using quantikine-high sensitivity ELISA kits from Bio-Techne Ltd. (R&D Systems). The detailed method was reported previously [35].

#### Whole blood stimulation

Whole blood for each participant was cultured using RPMI 1640 media (Sigma, Catalog# R8758) and stimulated for 24 h by TLR -2, -3 and -4 ligands, which are heat-killed Listeria monocytogenes (10^8^ cells/mL) (Invivogen, Catalog# tlrl-hklm), polyriboinosinic polyribocytidylic acid (10 µg/mL) (Invivogen, Catalog# tlrl-pic) and lipopolysaccharide (1 µg/mL) (Invivogen, Catalog# tlrl-eklps), respectively along with an untreated group for every participant as a control. The supernatants collected were tested for IL-6, IL-8, IL-10 and TNF-α levels. Principal component analysis (PCA) was performed on the data obtained from the whole blood stimulations to extract the best component values as a measure of TLR-2, -3 and -4 activities as previously reported [35].

#### DNA extraction and genotyping

Blood for DNA extraction was collected separately in another 10 mL EDTA tube for every participant, and DNA was extracted from individual samples using PAXgene blood DNA kits (Catalog # 761133; Qiagen). Genotyping was performed by Atlas Biolabs in Berlin, Germany on 288 iRELATE participants (194 controls and 94 patients) who provided consent, of which 281 participants passed the QC.

### DNA data processing

The Axiom Precision Medicine Research Array (PMRA) R3 platform was used for genotyping. The genotype data obtained was phased using Eagle v2.4 and imputed through Minimac4 v1.6.8 using the 1000 Genomes Phase III v5 reference panel [37] to obtain nearly 7.2 million SNPs. SNPs were removed if they failed any of the following QC metrics: minor allele frequency (MAF) < 0.1%, SNP missingness ≥ 5%, Hardy–Weinberg equilibrium p ≤ 1 ×10^-6^ or imputation quality score (INFO) < 0.9.

### Cognitive assessment

Full-scale IQ (FSIQ) was estimated with the abbreviated version of the Wechsler Adult Intelligence Scale, 3rd edition (WAIS-III) [38], which included subtests such as vocabulary, digit symbol coding, similarities, block design, and matrix reasoning. Episodic memory was evaluated through the Wechsler logical memory subtest and the CANTAB paired associates learning (PAL) task, focusing on total errors. Working memory was assessed using the Wechsler letter-number sequencing (LNS) subtest. Social cognition was measured with the Reading the Mind in the Eyes Test (RME). The detail list of cognition tasks and their assessment is included in our previously published paper [35].

### Statistical analysis and quality control

A Mann-Whitney non-parametric t-test was used to assess cytokine and cognition data and these were further subjected to Bonferroni corrections. In the case of cytokines, we assessed four of them so the new p-value cut-off is (0.05/4 = 0.013). We included six cognition variables, therefore, the new p-value cut-off is (0.05/6 = 0.008). ELISA data was managed and curated using Microsoft Excel. GraphPad Prism V.10.0.0 was employed to visualise the data.

Plink V.1.9 [39] facilitated quality control and analysis of genotyping data. Before further analysis, Mind-0.1 (exclude individuals with more than 10% missing genotype data), Geno-0.1 (exclude SNPs with more than 10% missing genotype data), MAF-0.01 (exclude SNPs with less than 1% minor allele frequency), and HWE-1 ×10-6 (Hardy-Weinberg Equilibrium) quality control filters were applied. SNPs at *TLR2* and *TLR4* were extracted within a window of 10,000 base pairs (bps) upstream and 1000 bps downstream of each gene based on the GRCh37 genome build. These extracted SNPs were tested for association using linear regression (additive model) [39], with phenotypes such as plasma cytokine levels and cognitive measures. The analyses were controlled for age, sex, and body mass index (BMI). Results underwent a 5% false discovery rate (FDR) correction via the “adjust” function in Plink, indicated by q-values. Subsequently, these results were clumped using the “clump” function. This function groups SNPs within a specific window based on how close they are to the “Index SNP” and likely to be inherited together, based on the linkage disequilibrium (LD) provided (>50% in this study). An ‘index SNP’ is the SNP with the lowest significance in the list. Specific clumping parameters such as “clump-p1” – 0.05 (significance threshold of index SNP), “clump-p2” – 0.05, and “clump-r2” – 0.5 (LD threshold) in Plink to identify index SNPs from the analysis. Significantly associated index SNPs that passed the FDR correction were reported. Allele frequencies and power calculations are present in Supplementary Tables 3 and 4 respectively.

## Results

### Demographics

Out of 288 genotyped participants, the study focused on 281 individuals (190 healthy controls and 91 clinically stable patients) who met the quality control criteria outlined in the methods section. These participants are part of the original previously reported iRELATE cohort [35]. Demographic and clinical characteristics are detailed in Table 1. The demographic data indicate that patients in our cohort were older, had a higher body mass index (BMI), and exhibited a higher incidence of depression compared to the controls (*p* < 0.001). Patients exhibited elevated circulating concentrations of IL-6 (*p* = 0.013) and IL-8 (*p* = 0.005) compared to the controls, while no significant differences were noted for IL-10 and TNF-α. Additionally, patients demonstrated a significantly lower full-scale IQ (*p* < 0.0001) with reduced performance across all five cognition tasks from the CANTAB battery (*p* < 0.0001) compared to the control group. The significant findings for cytokines and cognition remained valid after applying Bonferroni corrections, with p-value thresholds of *p* = 0.013 and *p* = 0.008, respectively.

**Table 1:** Clinical, cognitive and demographic data.

Total (N = 281)	Healthy controls (HC) (*N* = 190)		Schizophrenia (SZ) (*N* = 91)		HC vs. SZ
	Mean	Std. Dev.	Mean	Std. Dev.	*P* value
**Sex (M/F)**	109/81		59/32		0.25
**Age**	36.2	12.3	43.6	11	**<0.0001**
**BMI**	24.5	3.7	29.4	5.1	**<0.0001**
**Duration of illness (Years)**	-	-	17.2	10.1	
**HAM-D (17 items)**	2.8	2.8	4.9	4.9	**<0.001**
**Antipsychotics Olanzapine equivalent dose (mg/day)**			17.44	24.56	-
**PANSS**					
**Positive score**	-	-	8.9	2.9	-
**Negative score**	-	-	9.6	3.5	-
**General score**	-	-	21.4	4.9	-
**Total score**	-	-	39.9	9.1	-
**Plasma Cytokines (pg/mL)**					
**IL-6**	1.5	1.6	3.3	5.4	**0.013**
**IL-8**	3.8	2.5	5.8	3.5	**0.005**
**IL-10**	0.9	0.4	1.2	1.4	0.56
**TNF-α**	0.2	0.3	0.8	4.5	0.68
**Cognition tasks**					
**FSIQ**	112.5	16.2	93.8	16.9	**<0.0001**
**DSC**	77.1	15	54.3	17	**<0.0001**
**LM**	45.4	10	32.3	11.3	**<0.0001**
**PAL**	9.4	10.4	27.2	21.5	**<0.0001**
**LNS**	11.1	2.7	8.3	3.1	**<0.0001**
**RME**	27.3	4.1	22.8	5	**<0.0001**

*N* Sample size, *Std. Dev.* standard deviation, *M/F* Male/Female, *BMI* Body mass index, *HAM-D* Hamilton rating scale for depression, *pg/mL* picograms/millilitre, *IL* Interleukin, *TNF* Tumor necrosis factor, *FSIQ* full scale intelligent quotient, *DSC* digit symbol coding, *PAL* paired associates learning, *LM* Logical memory, *LNS* letter number sequencing, *RME* reading the mind in the eyes, *PANSS* Positive and negative syndrome scale, *Bold* Significant.

Non-parametric t-test were used to compare the differences between the groups.

### Greater TLR activity in patients

PCA was performed on the cytokine levels for each treatment [35]. The component with the highest eigenvalue was extracted as regression coefficients (Z-scores) for each TLR-stimulated group. These scores represented the activity of TLRs. Z-scores for TLR2 and TLR4 were extracted; however, scores for TLR3 could not be obtained due to an insufficient sample size, as several samples had IL-10 levels below the detection limit for TLR3 stimulation. Therefore, PCA was not suitable in this instance.

**Fig. 1:**
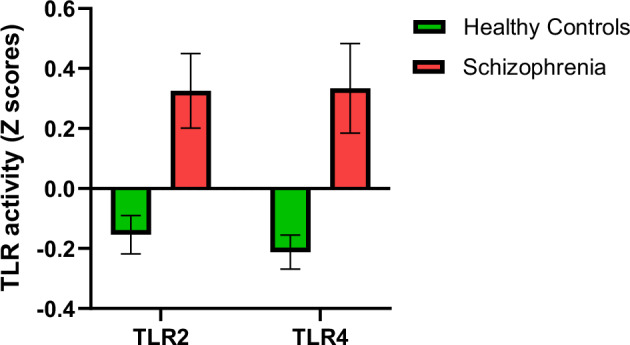
TLR activity in patients and controls. TLR – Toll-like receptor. Data is in the form of Mean ± Standard error of mean. The Y-axis values are Z-scores.

Z-scores (Fig. 1) indicate that TLR2 activity was higher in patients (Mean ± SD, N: 0.33 ± 1.2, 88) than controls (-0.15 ± 0.87, 186). Similarly, TLR4 activity was greater in patients (0.33 ± 1.3, 80) compared to controls (-0.2 ± 0.6, 126).

### TLR SNPs are associated with plasma cytokine levels but not with cognition in patients

**Fig. 2:**
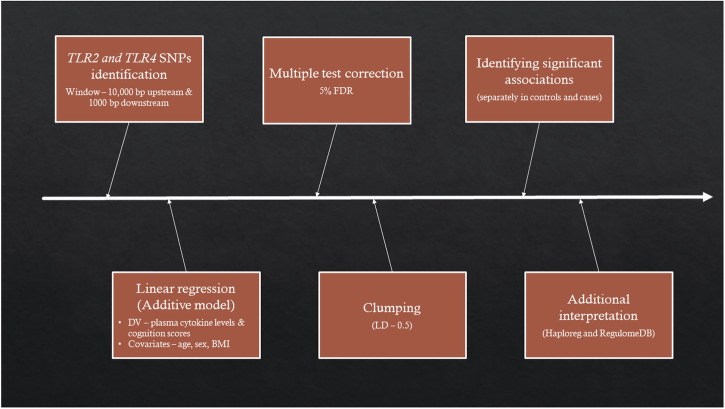
Work flow of the genotyping data analysis using *TLR2* and *TLR4* genes. *Bp* basepairs, *DV* dependent variable, *LD* linkage disequilibrium, *FDR* false discovery rate.

**Fig. 3:**
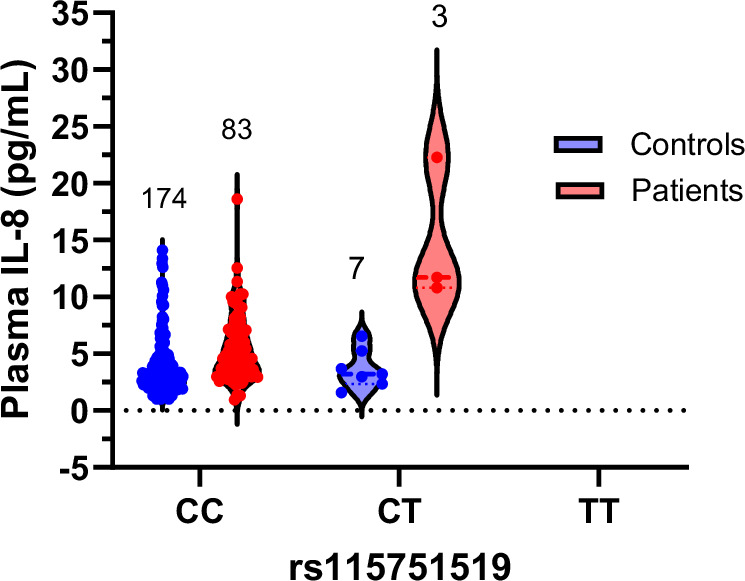
Plasma IL-8 levels in the participants with genotype of *TLR2* (rs115751519) in our cohort. Shown are plasma IL-8 levels (in pg/mL) for both controls (blue) and patients (red) for each of the three alleles CC, CT and TT for TLR2 rs115751519. Numbers on top indicate the number of people in each. Data is in the form of violin plot (all points).

Linear regressions followed by clumping was performed separately for patients and controls (Table 2). The work flow for this analysis is illustrated in Fig. 2. Using the 67 SNPs identified from the *TLR2* gene as the independent variable, with plasma cytokines as the dependent variable, while covarying for age, sex, and BMI. One SNP (rs115751519) was significantly associated (β = 11.9, *p*-value = 7.5 ×10-7 and *q*-value = 5 ×10-5) with increased plasma IL-8 levels (Fig. 3) in patients but not in controls (*p* = 0.86). No other significant associations were found between *TLR2* SNPs and IL-6, IL-10, or TNF-α in either patients or controls.

**Table 2:** Linear regressions between TLR SNPs and cytokines controlled for age, sex and BMI.

Gene (SNPs)	Phenotype (dependent variable) (pg/mL)	Index SNPs (clumping)	Alleles (Ref/Alt)	Variant type	Healthy Controls (*N* = 190)			Patients (N = 91)		
					Beta value	Significance	FDR 5% (q-value)	Beta value	Significance	FDR 5% (q-value)
*TLR2 (67)*	IL-8	rs115751519	C/T	Intron variant	0.6	0.385	0.87	**11.9**	**7.5 E-07**	**0.00005**
*TLR4 (87)*	IL-6	rs4986790	A/G	missense variant	**1.16**	**0.00028**	**0.008**	2.7	0.09	0.45
		rs1554973	T/C	3’ UTR variant	**0.59**	**0.0013**	**0.008**	1.3	0.15	0.55
		rs5030711	C/A	3’ UTR variant	**1.5**	**0.009**	**0.03**	-1.56	0.7	0.77
		rs10983755	G/A	intergenic variant (1.9 kb 5’)	-0.17	0.74	0.83	**9.4**	**0.00044**	**0.013**
		rs1927906	T/C	3’ UTR variant	**0.69**	**0.008**	**0.027**	**3.9**	**0.0025**	**0.036**
	Table 2. Linear regressions between TLR SNPs and cytokines controlled for age, sex and BMI.	rs5030711	C/A	3’ UTR variant	-0.19	0.17	0.66	**6.3**	**1.1 E-11**	**9.9 E-10**
		rs57863323	A/G	intergenic variant (6.7 kb 5’)	-0.13	0.12	0.62	**1.6**	**0.0024**	**0.029**

*TLR* Toll-like receptor, *SNP* Single-nucleotide polymorphism, *Ref* reference allele, *Alt* Alternate allele, *FDR* False discovery rate, *N* sample size, *UTR* untranslated region, *IL* interleukin, *TNF* Tumor necrosis factor.

Bold - significant associations (Index SNPs).

**Fig. 4:**
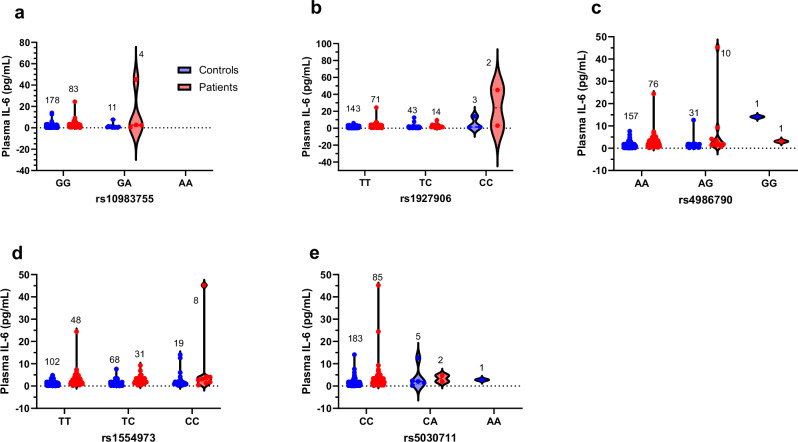
Plasma IL-6 levels in the participants with the genotypes of TLR4. **a** Shown are plasma IL-6 levels (in pg/mL) for both controls (blue) and patients (red) for each of the three alleles GG, GA and AA for TLR4 rs10983755, numbers on top of each plot indicate the number of people in each. Equivalent plots are shown in (**b**) for rs1927906 for TT, TC and CC alleles; (**c**) for rs4986790 for AA, AG and GG alleles; (**d**) for rs1554973 for TT, TC and CC alleles; and in (e) for rs5030711 for CC, CA and AA alleles. Data is in the form of violin plot (all points).

For the *TLR4* gene (87 SNPs), we identified five index SNPs that were significantly associated with increased IL-6 levels in both patients and controls (Fig. 4). Three of these SNPs (rs4986790, rs1554973, and rs5030711) were significant in controls, and one SNP (rs1927906) was significant in both groups. The last SNP, rs10983755 (Fig. 4a), showed a significant association with increased IL-6 levels in patients (β = 9.4, *p*-value = 0.0004 and *q*-value = 0.013) compared to controls (*p* = 0.73). The SNP, rs1927906 (Fig. 4b), demonstrated a significant association with increased IL-6 levels in both patients (β = 3.9, *p*-value = 0.0025 and *q*-value = 0.036) and controls (β = 0.7, *p*-value = 0.008 and *q*-value = 0.027). However, in controls, this SNP (rs1927906) was grouped (as a result of “clumping”) under the index SNP, rs4986790 (Fig. 4c), which was significantly associated with IL-6 (β = 1.2, *p*-value = 0.00028 and *q*-value = 0.008) in controls but not in patients (*p* = 0.09). Similarly, rs1554973 (Fig. 4d) and rs5030711 (Fig. 4e) were associated with increased IL-6 levels in controls (β = 0.6, *p*-value = 0.0013 and *q*-value = 0.008; β = 1.5, *p*-value = 0.009 and *q*-value = 0.03, respectively) but not in patients (*p* = 0.16, *p* = 0.7, respectively).

**Fig. 5:**
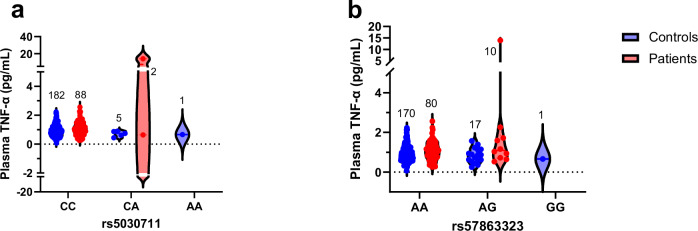
Plasma TNF-α levels with the genotypes of TLR4. Shown are plasma TNF-α levels (in pg/mL) for both controls (blue) and patients (red) for each of the three alleles CC, CA and AA for TLR4 rs5030711 in (**a**) and for TLR4 rs57863323 for each of the three alleles AA, AG and GG in (**b**). Numbers on top of each plot indicate numbers of people in each. Data is in the form of a violin plot (all points).

Additionally, seven significant SNPs were found to be associated with plasma TNF-α levels. After clumping, two SNPs (rs5030711, rs57863323) were identified (Fig. 5a, b). They were significantly associated with increased plasma TNF-α levels (β = 6.3, *p*-value = 1.1 ×10-11 and *q*-value = 9.9 ×10-10; β = 1.6, *p*-value = 0.0024 and *q*-value = 0.029, respectively).

In our previous study [35], we observed an association between TLR activity and cognition which was mediated by cytokines. Therefore, we investigated whether TLR genotypes are associated with cognition. However, we found no significant associations (q-value < 0.05) between *TLR2* and *TLR4* SNPs and full scale intelligent quotient, digit symbol coding task, paired associates learning task, logical memory, letter number sequencing task and social cognition (RME). The results for associations, including their multiple testing corrections (excluding their covariate data) for every SNP for controls and patients, are present in Supplementary Tables 1 and 3, and Supplementary Tables 2 and 4, respectively.

## Discussion

The literature and our previous study indicate that individuals with schizophrenia show elevated plasma cytokine levels and TLR activity [12, 35, 40]. It is also well established that TLR activation promotes cytokine synthesis [41]. This raises the question: Why are TLRs more active in patients? To explore this question, we investigated TLR genes. To the best of our knowledge, this is the first study to examine all common SNPs at the *TLR2* and *TLR4* genes that met the necessary SNP quality criteria and their association with circulating cytokine levels and cognition in patients.

From the plasma, we found elevated cytokine levels and higher TLR2 and TLR4 activity from stimulation studies in our patient group. Therefore, we investigated whether SNPs at the *TLR2* and *TLR4* genes affect cytokine levels. The literature reports that multiple SNPs at *TLR2* and *TLR4* genes are associated with an increased risk of schizophrenia [20, 21, 36, 42]. Our results indicate that rs115751519 (C > T), an intron variant of *TLR2*, was associated with increased plasma IL-8 levels in patients. *TLR4* SNP rs10983755 (G > A), an intergenic variant (1.9 kilobases 5’ of *TLR4*), was associated with increased plasma IL-6 levels in patients. Since it is located in the non-coding region and little information is available, using HaploReg V4.2 [43], we identified SNPs (rs7869402 and rs79895571) in strong linkage disequilibrium (LD) with our SNP. They are located in the 3’UTR region of the *TLR4* gene and have R^2^ values of 0.94 and 0.88, respectively. Further investigation using HaploReg and RegulomeDB V2.2 [44] showed that these two SNPs overlap chromatin states associated with active enhancers (Enh and EnhG) and transcription regions (TSSAFlnk and TxFlnk) in primary monocytes and neutrophils. This suggests that these SNPs might play a role in regulating other genes and immune function. Specifically, rs7869402, located in a functional regulatory region, binds transcription factors (TFs) such as FOS and STAT3, which play an active role in TLR4 signalling [45].

The SNP, rs1927906 (T > C), is a *TLR4* 3’ UTR variant associated with increased IL-6 levels in patients and controls, and shows evidence of increased enhancer activity in monocytes, neutrophils and B-lymphocytes. The alternate allele is also shown to increase the binding affinity of TFs, such as AP-1 and Ets, involved in immune cell development and TLR regulation [46], and decrease the binding affinity with Pou2f2 significantly, which is involved in B-cell differentiation and regulates gene expression in neutrophils [47]. Moreover, another *TLR4* 3’ UTR variant, which we found to be linked with increased IL-6 levels in controls, is rs1554973 (T > C), which shows evidence of enhancer and transcription (TssAFlnk) activity in monocytes and neutrophils.

Similarly, we found rs5030711 (C > A; *TLR4* SNP), a synonymous (or 3’UTR) variant, associated with increased TNF-α levels in patients and IL-6 levels in controls. It reportedly regulates the gene enhancer regions in monocytes and neutrophils and is predicted to alter the binding of TFs. Notably, the alternate allele slightly reduces NF-κB’s (significant role in the TLR pathway leading to cytokine synthesis) binding affinity C to A. Another *TLR4* SNP significantly associated in our study is rs57863323 (A > G), an intergenic variant (6.7 kilobases 5’ of *TLR4*) that reportedly regulates the enhancer region of neutrophils. The alternate allele is linked to a significant reduction in the binding affinity of TFs, such as Arid5b (A to G; this TF plays a role in the differentiation of B-lymphocyte progenitors) and Osf2 (A to G; this TF plays a role in osteoblast differentiation) and it significantly increases the binding affinity of HDAC2 (A to G; this TF plays a role in transcription regulation, specifically as a repressor). The HDAC2 role is also observed in inflammatory suppression [48].

In the literature to date, the aforementioned significant SNPs have not been investigated in relation to schizophrenia. Therefore, either their functional effects or their association with the risk of schizophrenia is unclear. One exception is rs4986790 (*TLR4* SNP), which was previously reported to be associated with the risk of schizophrenia [22]. Interestingly, this SNP is significantly associated with an increase in plasma IL-6 levels in our cohort but not the schizophrenia group. It is not known why such a discrepancy exists between our studies; however, investigating larger cohorts can provide clarity.

Interestingly, unlike previous reports in the literature, where *TLR2* SNPs (rs3804099, rs3804100) were associated with poor concentration in schizophrenia [36], we found no associations between *TLR2* and *TLR4* genes and cognitive measures. Factors such as ethnicity and patient disease state might play a role in this, as Kang and colleagues’ study included inpatients of Korean ethnicity [36], whereas our cohort was comprised of stable outpatients of Irish origin.

One of the biggest strengths of this paper is that we investigated every SNP in the *TLR2* and *TLR4* genes in relation to plasma cytokine levels, and analysing the entire gene in this manner has never been done before in a schizophrenia cohort. Another strength is that we addressed the possible SNPs in LD, which could influence gene regulation and transcription factors that may have resulted in increased cytokine levels. Some limitations in this study include the minor allele frequency (MAF), which was set at >1% to capture both standard and rare *TLR* variants. However, such rare variants require larger sample sizes. Although a sample size of 281, including 91 patients, might be considered reasonable for a study investigating clinical, biological, and behavioural data, it is deemed modest for genotyping research. Particularly given the MAF > 1% in our study, an ideal sample size for patients and controls would be 10–20 times our current sample size [49], which can be seen in UK Biobank data (as of 2021) comprising ~1400 schizophrenia patients [50].

In conclusion, our findings show that *TLR2* and *TLR4* SNPs might indirectly increase cytokine levels. This effect is highly significant in the case of patients. This indicates that patients carrying particular genotypes may be predisposed to elevated cytokine levels. However, these variants are not associated with cognition.

## Supplementary information

Supplementary Table 1

Supplementary Table 2

Supplementary Table 3

Supplementary Table 4

## Data Availability

Datasets as well as analyses for this study not supplied in manuscript or supplementary data will be available from the corresponding author upon reasonable request.
